# Supplementary material for: Nuclear basket proteins Nup2 and Mlp1 drive heat shock–induced 3D genome restructuring downstream of transcriptional activation
Source: J Biol Chem. 2025 Aug 6;301(9):110568. doi: 10.1016/j.jbc.2025.110568 (PMC12455135; doi:10.1016/j.jbc.2025.110568)
Supplement: Supplemental Figures [file mmc1.pptx]

## Slide 1
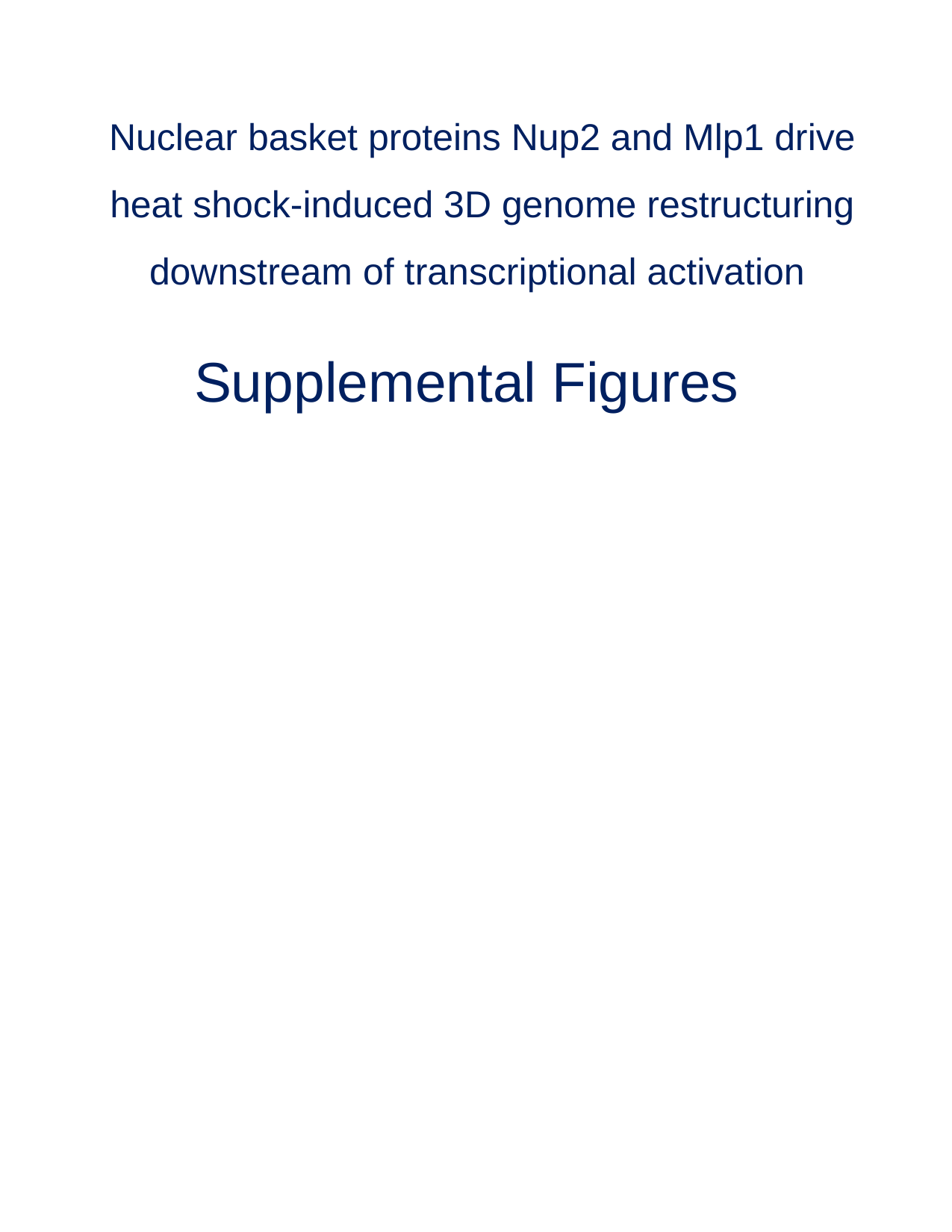

Nuclear basket proteins Nup2 and Mlp1 drive heat shock-induced 3D genome restructuring downstream of transcriptional activation
# Supplemental Figures

## Slide 2
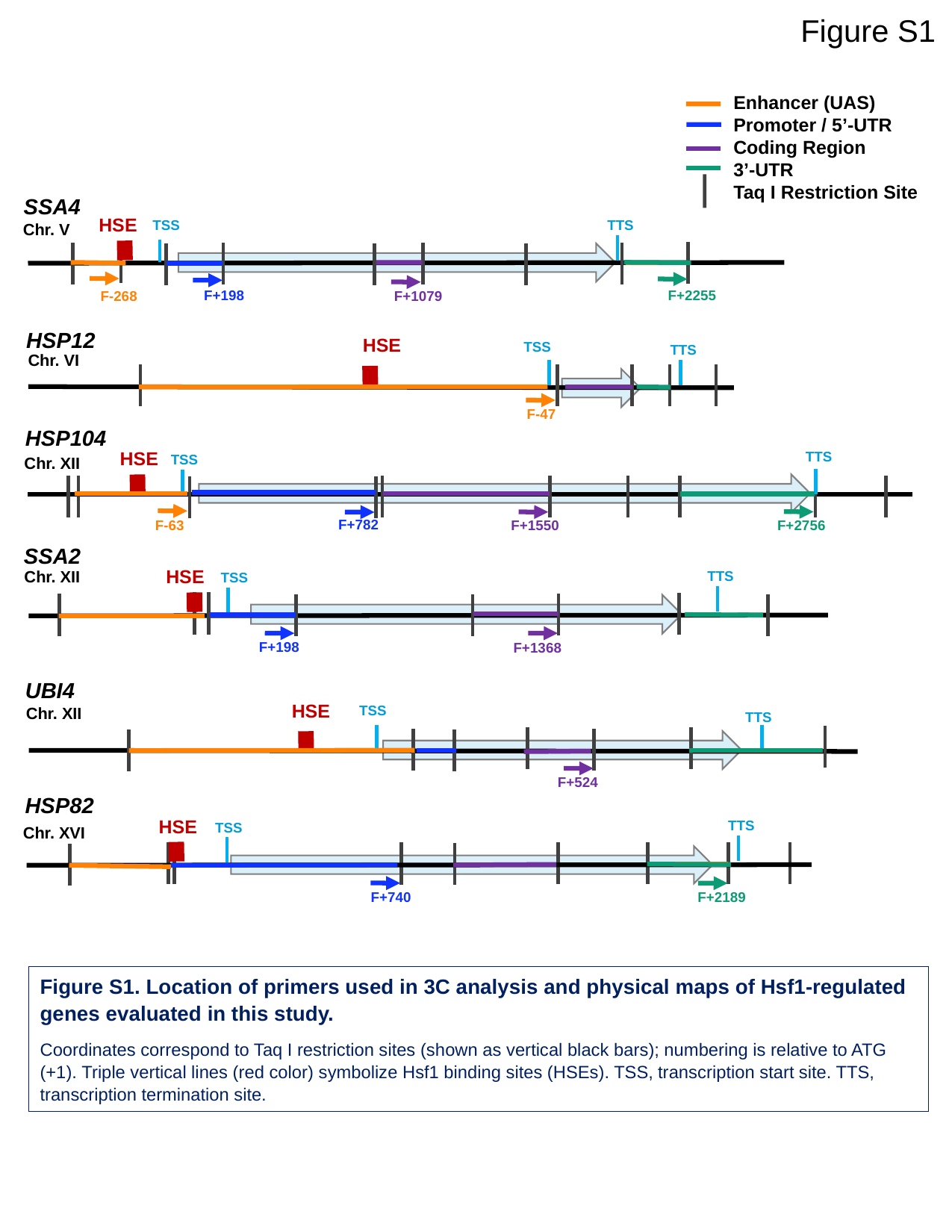

Figure S1
Enhancer (UAS)
Promoter / 5’-UTR
Coding Region
3’-UTR
Taq I Restriction Site
SSA4
HSE
TTS
TSS
F+198
F+1079
F-268
Chr. V
F+2255
HSP12
HSE
TSS
TTS
F-47
Chr. VI
HSP104
HSE
TTS
TSS
F+782
F-63
F+2756
F+1550
Chr. XII
SSA2
HSE
TTS
TSS
 F+198
F+1368
Chr. XII
UBI4
HSE
TSS
TTS
F+524
Chr. XII
HSP82
HSE
TTS
TSS
F+2189
F+740
Chr. XVI
Figure S1. Location of primers used in 3C analysis and physical maps of Hsf1-regulated genes evaluated in this study.
Coordinates correspond to Taq I restriction sites (shown as vertical black bars); numbering is relative to ATG (+1). Triple vertical lines (red color) symbolize Hsf1 binding sites (HSEs). TSS, transcription start site. TTS, transcription termination site.

## Slide 3
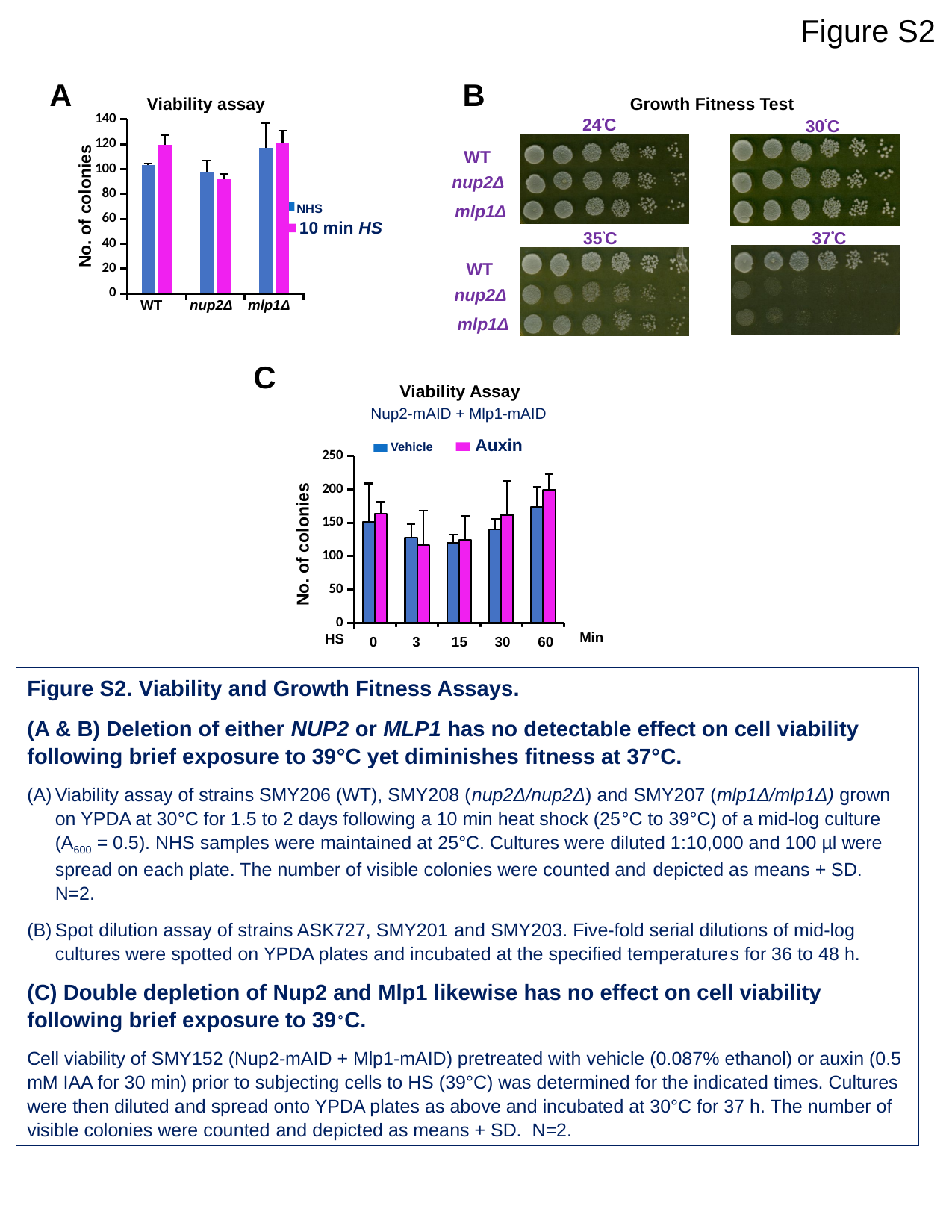

# Figure S2
A
B
 Viability assay
No. of colonies
 NHS
 10 min HS
 WT nup2Δ mlp1Δ
 Growth Fitness Test
### Chart
| Category | NHS | 10m HS |
|---|---|---|
| Wild type | 103.0 | 119.5 |
| nup2∆ | 97.5 | 92.0 |
| mlp1∆ | 117.0 | 121.5 |24°C
30°C
WT
nup2Δ
mlp1Δ
37°C
35°C
WT
nup2Δ
mlp1Δ
C
### Chart
| Category | Vehicle | 0.5mM IAA |
|---|---|---|
| 0m | 151.0 | 164.0 |
| 3m | 128.0 | 116.5 |
| 15m | 120.0 | 125.0 |
| 30m | 140.5 | 162.0 |
| 60m | 173.5 | 199.5 | Viability Assay
Nup2-mAID + Mlp1-mAID
 Auxin
 Vehicle
 No. of colonies
 Min
 HS
0 3 15 30 60
Figure S2. Viability and Growth Fitness Assays.
(A & B) Deletion of either NUP2 or MLP1 has no detectable effect on cell viability following brief exposure to 39°C yet diminishes fitness at 37°C.
Viability assay of strains SMY206 (WT), SMY208 (nup2Δ/nup2Δ) and SMY207 (mlp1Δ/mlp1Δ) grown on YPDA at 30°C for 1.5 to 2 days following a 10 min heat shock (25°C to 39°C) of a mid-log culture (A600 = 0.5). NHS samples were maintained at 25°C. Cultures were diluted 1:10,000 and 100 µl were spread on each plate. The number of visible colonies were counted and depicted as means + SD.  N=2.
Spot dilution assay of strains ASK727, SMY201 and SMY203. Five-fold serial dilutions of mid-log cultures were spotted on YPDA plates and incubated at the specified temperatures for 36 to 48 h.
(C) Double depletion of Nup2 and Mlp1 likewise has no effect on cell viability following brief exposure to 39∘C.
Cell viability of SMY152 (Nup2-mAID + Mlp1-mAID) pretreated with vehicle (0.087% ethanol) or auxin (0.5 mM IAA for 30 min) prior to subjecting cells to HS (39°C) was determined for the indicated times. Cultures were then diluted and spread onto YPDA plates as above and incubated at 30°C for 37 h. The number of visible colonies were counted and depicted as means + SD.  N=2.

## Slide 4
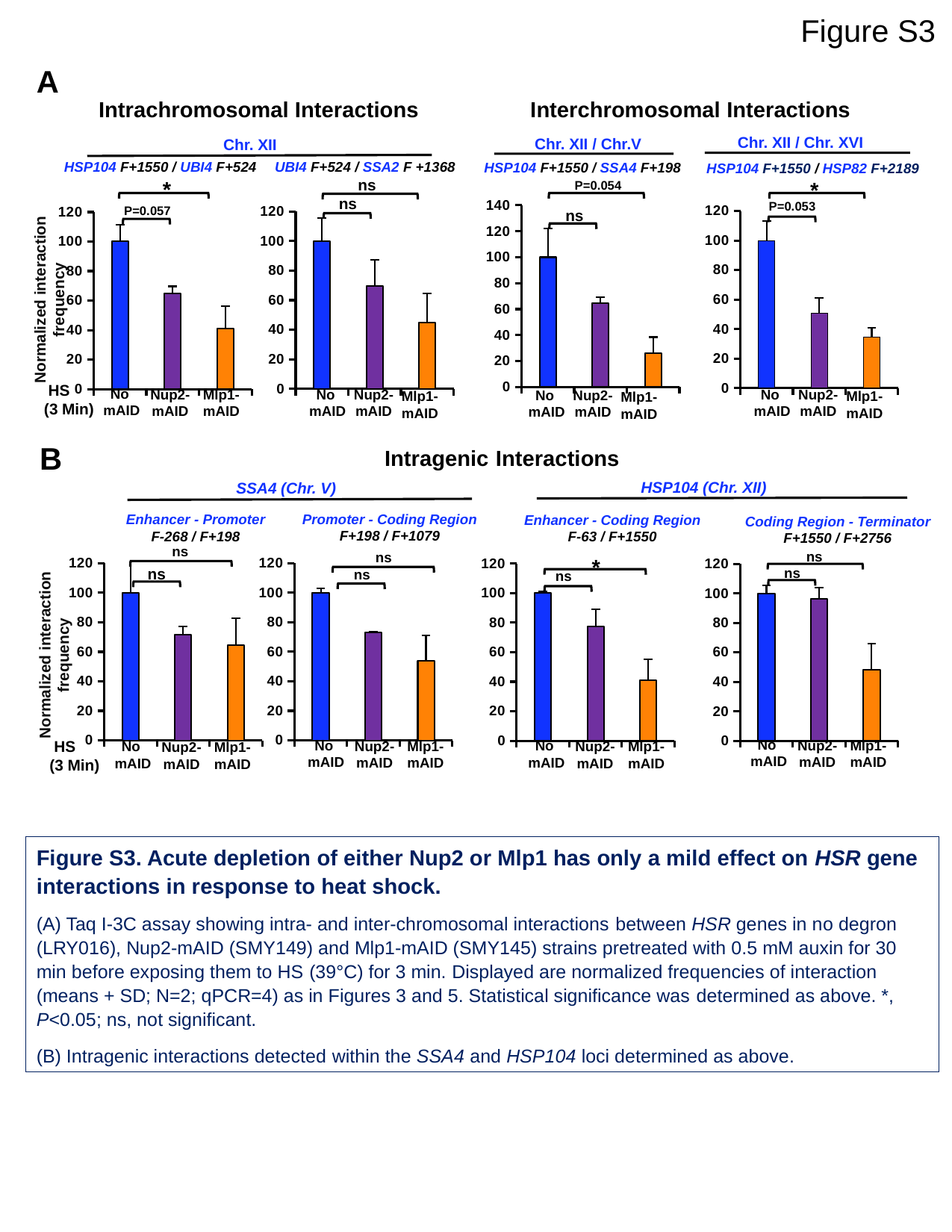

# Figure S3
A
Intrachromosomal Interactions
Interchromosomal Interactions
Chr. XII / Chr. XVI
Chr. XII / Chr.V
Chr. XII
HSP104 F+1550 / UBI4 F+524
UBI4 F+524 / SSA2 F +1368
### Chart
| Category | 3m HS |
|---|---|
| Untag | 100.0 |
| Nup2 degron | 69.6212177439826 |
| Mlp1 degron | 44.7938349971961 |
### Chart
| Category | 3m HS |
|---|---|
| Untag | 100.0 |
| Nup2 degron | 64.85578963029147 |
| Mlp1 degron | 41.15836993247535 |Normalized interaction frequency
HSP104 F+1550 / SSA4 F+198
HSP104 F+1550 / HSP82 F+2189
ns
*
P=0.054
*
ns
### Chart
| Category | 3m HS |
|---|---|
| Untag | 100.0 |
| Nup2 degron | 64.5495000706558 |
| Mlp1 degron | 25.840412850109907 |P=0.053
P=0.057
### Chart
| Category | 3m HS |
|---|---|
| Untag | 100.0 |
| Nup2 degron | 50.59268794130672 |
| Mlp1 degron | 34.60221608137255 |ns
No
mAID
No
mAID
Nup2-
mAID
Nup2-
mAID
Mlp1-
mAID
No
mAID
Nup2-
mAID
No
mAID
Nup2-
mAID
Mlp1-
mAID
Mlp1-
mAID
Mlp1-
mAID
 HS
 (3 Min)
B
Intragenic Interactions
HSP104 (Chr. XII)
SSA4 (Chr. V)
Promoter - Coding Region
F+198 / F+1079
Enhancer - Promoter
F-268 / F+198
Enhancer - Coding Region
F-63 / F+1550
Coding Region - Terminator
F+1550 / F+2756
ns
ns
ns
ns
ns
*
### Chart
| Category | 3m HS |
|---|---|
| Untag | 100.0 |
| Nup2 degron | 73.11319628322579 |
| Mlp1 degron | 53.78517715231193 |
### Chart
| Category | 3m HS |
|---|---|
| Untag | 100.0 |
| Nup2 degron | 71.50423285862368 |
| Mlp1 degron | 64.49714061196558 |
### Chart
| Category | 3m HS |
|---|---|
| Untag | 100.0 |
| Nup2 degron | 77.4965669515828 |
| Mlp1 degron | 40.97157243861724 |
### Chart
| Category | 3m HS |
|---|---|
| Untag | 99.99999999999999 |
| Nup2 degron | 96.34891052365828 |
| Mlp1 degron | 48.19342718935263 |ns
ns
Normalized interaction frequency
No
mAID
No
mAID
Nup2-
mAID
Mlp1-
mAID
No
mAID
Nup2-
mAID
Mlp1-
mAID
No
mAID
Nup2-
mAID
Mlp1-
mAID
Nup2-
mAID
Mlp1-
mAID
 HS
 (3 Min)
Figure S3. Acute depletion of either Nup2 or Mlp1 has only a mild effect on HSR gene interactions in response to heat shock.
(A) Taq I-3C assay showing intra- and inter-chromosomal interactions between HSR genes in no degron (LRY016), Nup2-mAID (SMY149) and Mlp1-mAID (SMY145) strains pretreated with 0.5 mM auxin for 30 min before exposing them to HS (39°C) for 3 min. Displayed are normalized frequencies of interaction (means + SD; N=2; qPCR=4) as in Figures 3 and 5. Statistical significance was determined as above. *, P<0.05; ns, not significant.
(B) Intragenic interactions detected within the SSA4 and HSP104 loci determined as above.

## Slide 5
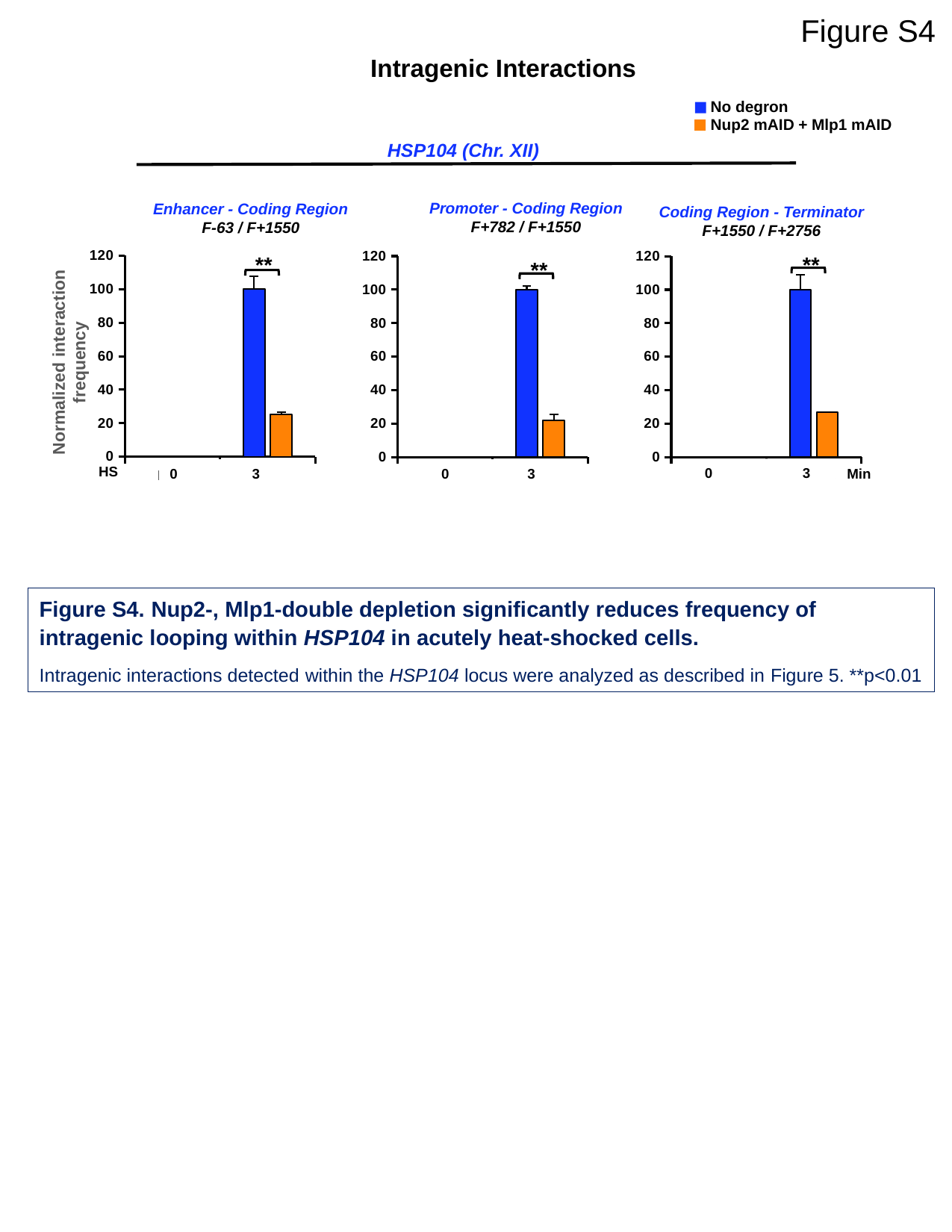

Figure S4
Intragenic Interactions
No degron
Nup2 mAID + Mlp1 mAID
HSP104 (Chr. XII)
Promoter - Coding Region
F+782 / F+1550
Enhancer - Coding Region
F-63 / F+1550
Coding Region - Terminator
F+1550 / F+2756
### Chart
| Category | Untag | Double tag |
|---|---|---|
| NHS | 0.012593480626324857 | 0.036938234413478696 |
| 3m HS | 100.0 | 25.286596655720714 |
### Chart
| Category | Untag | Double tag |
|---|---|---|
| NHS | 0.02330983976975188 | 0.01655946543032551 |
| 3m HS | 100.0 | 22.064893208920864 |
### Chart
| Category | Untag | Double tag |
|---|---|---|
| NHS | 0.0049124129643686835 | 0.0038426959967989968 |
| 3m HS | 100.0 | 26.86564902150974 |**
**
**
Normalized interaction frequency
HS
0 3
Min
0 3
0 3
Figure S4. Nup2-, Mlp1-double depletion significantly reduces frequency of intragenic looping within HSP104 in acutely heat-shocked cells.
Intragenic interactions detected within the HSP104 locus were analyzed as described in Figure 5. **p<0.01

## Slide 6
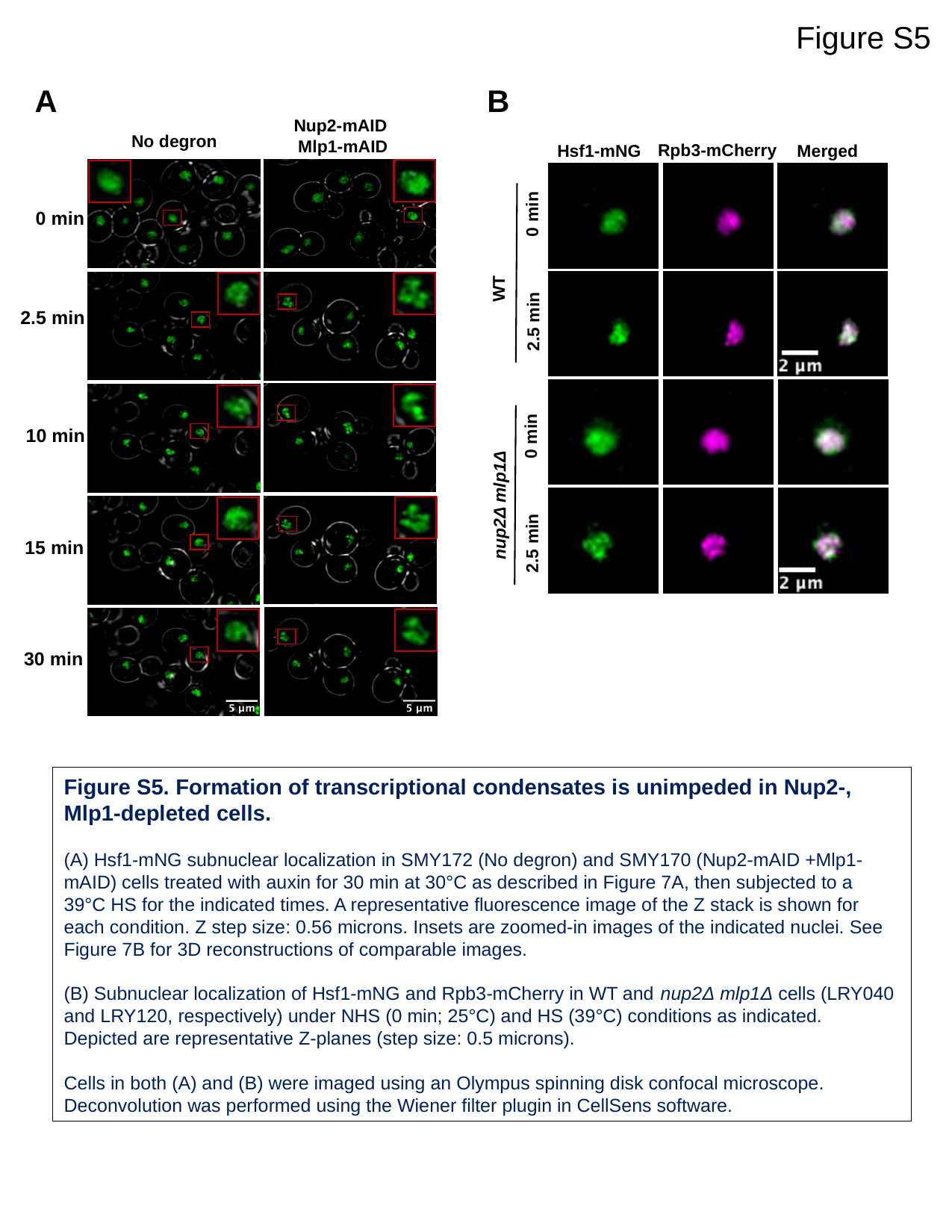

Figure S5
A
B
Nup2-mAID
 Mlp1-mAID
No degron
Rpb3-mCherry
Hsf1-mNG
Merged
0 min
2.5 min
0 min
2.5 min
0 min
WT
2.5 min
10 min
nup2Δ mlp1Δ
15 min
30 min
Figure S5. Formation of transcriptional condensates is unimpeded in Nup2-, Mlp1-depleted cells.
(A) Hsf1-mNG subnuclear localization in SMY172 (No degron) and SMY170 (Nup2-mAID +Mlp1-mAID) cells treated with auxin for 30 min at 30°C as described in Figure 7A, then subjected to a 39°C HS for the indicated times. A representative fluorescence image of the Z stack is shown for each condition. Z step size: 0.56 microns. Insets are zoomed-in images of the indicated nuclei. See Figure 7B for 3D reconstructions of comparable images.
(B) Subnuclear localization of Hsf1-mNG and Rpb3-mCherry in WT and nup2Δ mlp1Δ cells (LRY040 and LRY120, respectively) under NHS (0 min; 25°C) and HS (39°C) conditions as indicated. Depicted are representative Z-planes (step size: 0.5 microns).
Cells in both (A) and (B) were imaged using an Olympus spinning disk confocal microscope. Deconvolution was performed using the Wiener filter plugin in CellSens software.

## Slide 7
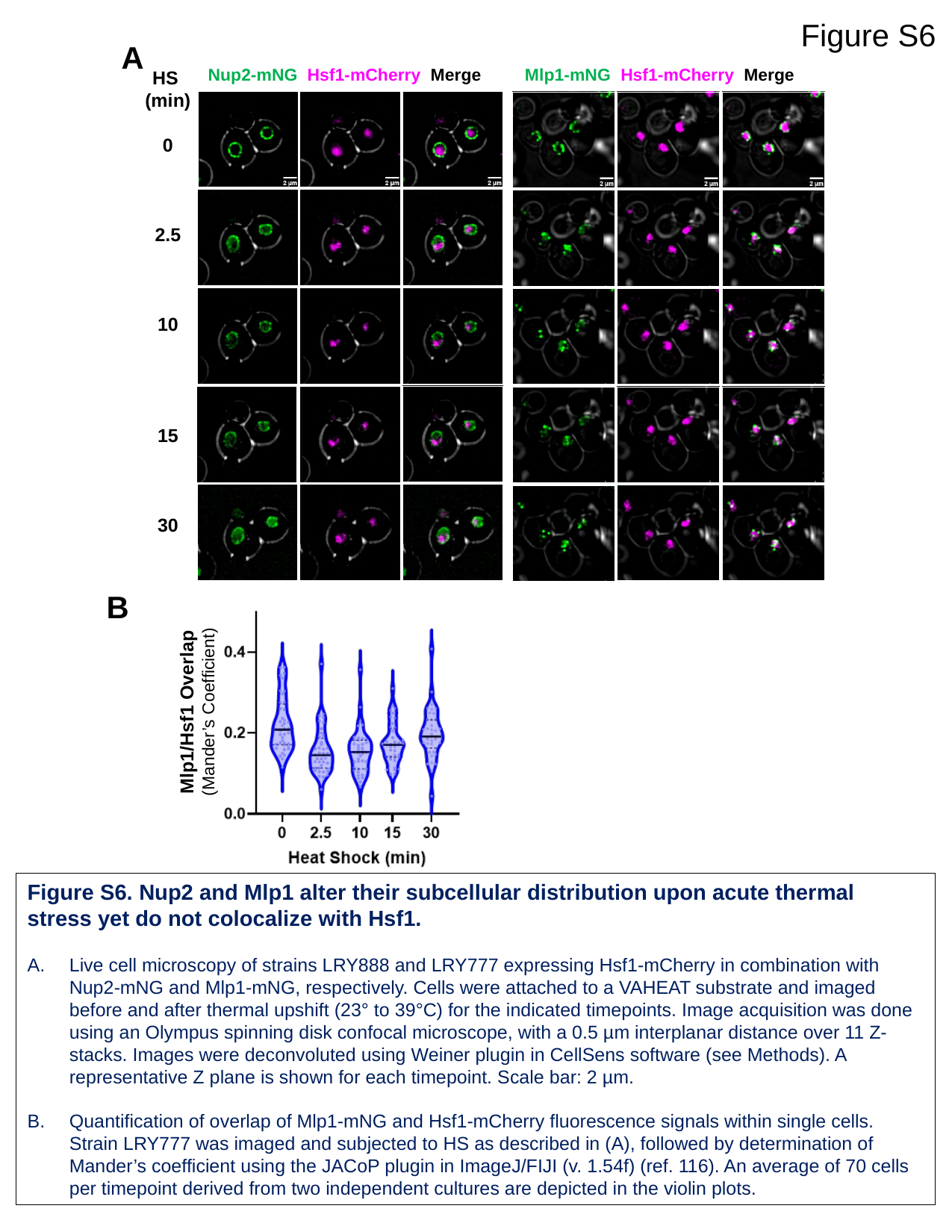

Figure S6
A
Nup2-mNG Hsf1-mCherry Merge Mlp1-mNG Hsf1-mCherry Merge
HS
(min)
0
2.5
10
15
30
B
Mlp1/Hsf1 Overlap
(Mander’s Coefficient)
Figure S6. Nup2 and Mlp1 alter their subcellular distribution upon acute thermal stress yet do not colocalize with Hsf1.
Live cell microscopy of strains LRY888 and LRY777 expressing Hsf1-mCherry in combination with Nup2-mNG and Mlp1-mNG, respectively. Cells were attached to a VAHEAT substrate and imaged before and after thermal upshift (23° to 39°C) for the indicated timepoints. Image acquisition was done using an Olympus spinning disk confocal microscope, with a 0.5 µm interplanar distance over 11 Z-stacks. Images were deconvoluted using Weiner plugin in CellSens software (see Methods). A representative Z plane is shown for each timepoint. Scale bar: 2 µm.
Quantification of overlap of Mlp1-mNG and Hsf1-mCherry fluorescence signals within single cells. Strain LRY777 was imaged and subjected to HS as described in (A), followed by determination of Mander’s coefficient using the JACoP plugin in ImageJ/FIJI (v. 1.54f) (ref. 116). An average of 70 cells per timepoint derived from two independent cultures are depicted in the violin plots.
